# The cost-effectiveness of procalcitonin for guiding antibiotic prescribing in individuals hospitalized with COVID-19: part of the PEACH study

**DOI:** 10.1093/jac/dkae167

**Published:** 2024-06-06

**Authors:** Edward J D Webb, Daniel Howdon, Rebecca Bestwick, Natalie King, Jonathan A T Sandoe, Joanne Euden, Detelina Grozeva, Robert West, Philip Howard, Neil Powell, Mahableshwar Albur, Stuart Bond, Lucy Brookes-Howell, Paul Dark, Thomas Hellyer, Martin Llewelyn, Iain J McCullagh, Margaret Ogden, Philip Pallmann, Helena Parsons, David Partridge, Dominick Shaw, Tamas Szakmany, Stacy Todd, Emma Thomas-Jones, Enitan D Carrol, Bethany Shinkins, PEACH Study Group, Jonathan Sandoe, Enitan Carrol, Emma Thomas-Jones, Lucy Brookes-Howell, Josie Henley, Wakunyambo Maboshe, Philip Pallmann, Detelina Grozeva, Marcin Bargiel, Judith Evans, Edward Webb, Rebecca Bestwick, Daniel Howdon, Robert West, Colin Richman, Sarah Gerver, Russell Hope, Susan Hopkins, Margaret Heginbothom, Philip Howard, Jonathan Sandoe, Claire Berry, Georgina Davis, Vikki Wilkinson, Stacy Todd, Eleanor Taylor-Barr, Mary Brodsky, Jo Brown, Jenni Burns, Sharon Glynn, Alvyda Gureviciute, Megan Howard, Jennifer Kirkpatrick, Hannah Murphy, Emma Richardson, Deborah Scanlon, Claire Small, Graham Sweeney, Lisa Williams, Tamas Szakmany, Evelyn Baker, Yusuf Cheema, Jill Dunhill, Charlotte Killick, Charlie King, Simran Kooner, Swyn Lewis, Maxine Nash, Owen Richardson, Jemma Tuffney, Clare Westacott, Sarah Williams, David Partridge, Helena Parsons, Kay Cawthron, Yuen Kiu Tai, Thomas Newman, Megan Plowright, Helen Shulver, Anna Sivakova, Neil Powell, Freddie Ayliffe, Emma Darke, Eve Fletcher, Fiona Hammonds, Gladys Marquez, Leanne Welch, Stuart Bond, Jade Lee-Milner, Joseph Spencer, Mahableshwar Albur, Rodrigo Brandao, Joshua Hrycaiczuk, Jack Stanley, Martin Llewelyn, Elizabeth Cross, Daniel Hansen, Ethan Redmore, Abigail Whyte, Tom Hellyer, Iain McCullagh, Benjamin Brown, Michele Calabrese, Cameron Cole, Jessica DeSousa, Leigh Dunn, Stephanie Grieveson, Arti Gulati, Elizabeth Issac, Ruaridh Mackay, Fatima Simoes, Paul Dark, Elena Apatri, Bethan Charles, Helen Christensen, Alice Harvey, Diane Lomas, Melanie Taylor, Vicky Thomas, Danielle Walker, Dominick Shaw, Lucy Howard, Amelia Joseph, Saheer Sultan, Chikezie Knox-Macaulay, Margaret Ogden, Graham Prestwich, Ryan Hamilton

**Affiliations:** Leeds Institute for Health Sciences, University of Leeds, Leeds, UK; Leeds Institute for Health Sciences, University of Leeds, Leeds, UK; Leeds Institute for Health Sciences, University of Leeds, Leeds, UK; Leeds Institute for Health Sciences, University of Leeds, Leeds, UK; Healthcare Associated Infection Group, Leeds Institute of Medical Research, University of Leeds, Leeds, UK; Department of Microbiology, Leeds Teaching Hospitals NHS Trust, Leeds, UK; Centre for Trials Research, College of Biomedical and Life Sciences, Cardiff University, Cardiff, UK; Centre for Trials Research, College of Biomedical and Life Sciences, Cardiff University, Cardiff, UK; Leeds Institute for Health Sciences, University of Leeds, Leeds, UK; Healthcare Associated Infection Group, Leeds Institute of Medical Research, University of Leeds, Leeds, UK; NHS England North-East & Yorkshire, Leeds, UK; Pharmacy Department, Royal Cornwall Hospital, Royal Cornwall Hospitals NHS Foundation Trust, Truro TR1 3LJ, UK; Severn Infectious Sciences, Southmead Hospital, North Bristol NHS Trust, Bristol BS10 5NB, UK; Medicines Optimisation and Pharmacy Services, Pinderfields Hospital, Mid Yorkshire Teaching NHS Trust, Wakefield WF1 4DG, UK; Centre for Trials Research, College of Biomedical and Life Sciences, Cardiff University, Cardiff, UK; Division of Immunology, Faculty of Biology, Medicine and Health, Immunity to Infection and Respiratory Medicine, School of Biological Sciences, University of Manchester, Manchester M13 9PL, UK; Perioperative and Critical Care Department, Institute of Transplantation, Freeman Hospital, Newcastle upon Tyne Hospital NHS Foundation Trust, Newcastle upon Tyne NE7 7DN, UK; Faculty of Medical Sciences, Translational and Clinical Research Institute, Newcastle University, Newcastle upon Tyne NE2 4HH, UK; Global Health and Infection, Brighton and Sussex Medical School, University of Sussex, Brighton BN1 9PS, UK; Department of Infection Medicine, University Hospitals Sussex NHS Foundation Trust, Brighton, UK; Perioperative and Critical Care Department, Institute of Transplantation, Freeman Hospital, Newcastle upon Tyne Hospital NHS Foundation Trust, Newcastle upon Tyne NE7 7DN, UK; Faculty of Medical Sciences, Translational and Clinical Research Institute, Newcastle University, Newcastle upon Tyne NE2 4HH, UK; Public and Patient Involvement Representative, NIHR, London SW1A 2NS, UK; Centre for Trials Research, College of Biomedical and Life Sciences, Cardiff University, Cardiff, UK; Department of Microbiology, Laboratory Medicine, Northern General Hospital, Sheffield Teaching Hospital NHS Foundation Trust, Sheffield S5 7AU, UK; Department of Microbiology, Laboratory Medicine, Northern General Hospital, Sheffield Teaching Hospital NHS Foundation Trust, Sheffield S5 7AU, UK; Department of Respiratory Sciences, University of Leicester, Leicester, UK; Royal Gwent Hospital, Aneurin Bevan University Health Board, Newport, UK; Tropical and Infectious Disease Unit, The Royal Liverpool and Broadgreen University Hospitals NHS Trust, Liverpool, UK; Centre for Trials Research, College of Biomedical and Life Sciences, Cardiff University, Cardiff, UK; Department of Clinical Infection, Microbiology and Immunology, Institute of Infection, Veterinary and Ecological Sciences, University of Liverpool, Liverpool, UK; Leeds Institute for Health Sciences, University of Leeds, Leeds, UK; Division of Health Sciences, Warwick Medical School, University of Warwick, Coventry, UK

## Abstract

**Background:**

Many hospitals introduced procalcitonin (PCT) testing to help diagnose bacterial coinfection in individuals with COVID-19, and guide antibiotic decision-making during the COVID-19 pandemic in the UK.

**Objectives:**

Evaluating cost-effectiveness of using PCT to guide antibiotic decisions in individuals hospitalized with COVID-19, as part of a wider research programme.

**Methods:**

Retrospective individual-level data on patients hospitalized with COVID-19 were collected from 11 NHS acute hospital Trusts and Health Boards from England and Wales, which varied in their use of baseline PCT testing during the first COVID-19 pandemic wave. A matched analysis (part of a wider analysis reported elsewhere) created groups of patients whose PCT was/was not tested at baseline. A model was created with combined decision tree/Markov phases, parameterized with quality-of-life/unit cost estimates from the literature, and used to estimate costs and quality-adjusted life years (QALYs). Cost-effectiveness was judged at a £20 000/QALY threshold. Uncertainty was characterized using bootstrapping.

**Results:**

People who had baseline PCT testing had shorter general ward/ICU stays and spent less time on antibiotics, though with overlap between the groups’ 95% CIs. Those with baseline PCT testing accrued more QALYs (8.76 versus 8.62) and lower costs (£9830 versus £10 700). The point estimate was baseline PCT testing being dominant over no baseline testing, though with uncertainty: the probability of cost-effectiveness was 0.579 with a 1 year horizon and 0.872 with a lifetime horizon.

**Conclusions:**

Using PCT to guide antibiotic therapy in individuals hospitalized with COVID-19 is more likely to be cost-effective than not, albeit with uncertainty.

## Introduction

The COVID-19 pandemic has been a global health crisis, with millions of cases and fatalities worldwide. One of the critical issues that has emerged during the pandemic is the inappropriate use of antibiotics in the management of individuals with COVID-19, particularly in those hospitalized.^1^ Determining whether COVID-19 patients have a bacterial coinfection and who therefore may benefit from antibiotics is challenging, particularly because many of the frequently used biomarkers of infection, such as C-reactive protein (CRP), are often elevated in individuals with COVID-19.^2^ Inappropriate and excessive use of antibiotics can contribute to antimicrobial resistance (AMR), which can cause infections that are difficult or impossible to treat, and therefore interventions to support appropriate antibiotic prescribing decisions are needed.

Procalcitonin (PCT) is an inflammatory biomarker, measured in the blood, that rises when bacterial infection is present and falls in response to effective antimicrobial treatment. A Cochrane meta-analysis has demonstrated that PCT can guide antibiotic therapy in non-COVID-19 acute respiratory infections with reduced antibiotic exposure and improved survival.^3^ During the first wave of the COVID-19 pandemic in the UK, many hospitals introduced PCT testing to help diagnose bacterial coinfection in individuals with COVID-19 and guide antibiotic decision-making.^4^ This was at odds with US and UK national guidelines on the management of community-acquired pneumonia, which recommended against the use of PCT to guide antibiotic prescribing.^5,6^

The Procalcitonin Evaluation of Antibiotic use in COVID-19 Hospitalised patients (PEACH) study evaluated whether the use of PCT testing to guide antibiotic prescribing safely reduced antibiotic use among patients admitted to acute UK NHS hospitals with COVID-19.^7^ The study consisted of organization-level and individual patient-level analyses, both investigating the utility of PCT for guiding antibiotic prescribing. An initial survey of 148 (of 151; 98%) acute hospitals in England and Wales demonstrated increased use of PCT testing in emergency and acute admissions, which preceded development of the NICE guidance. The survey ascertained whether PCT testing was adopted during the pandemic and, if so, in which areas of the hospital, and factors relating to the test use and interpretation (e.g. cut-offs, testing algorithm).^4^ A retrospective analysis of organization-level data over time found that the introduction of PCT testing in emergency departments or acute medical admission units was associated with an initial, but non-sustained, reduction in total antibiotic use.^8^

The aim of this paper was to explore the cost-effectiveness of using PCT testing to guide antibiotic decisions in individuals hospitalized with COVID-19 based on a matched analysis of individual-level data collected from 11 UK NHS Trusts and Health Boards.

## Materials and methods

All analyses were performed in R version 4.3.1.

### Data

Retrospective individual-level data on patients hospitalized with COVID-19 were collected from 11 NHS acute hospital Trusts and Health Boards from England and Wales, some of which used PCT testing routinely in COVID-19 patients during the first wave of the pandemic and some of which did not. In line with the study protocol,^7^ patient characteristics such as age, gender, ethnicity and comorbidities were recorded, along with hospital admission and discharge dates, ICU admission and discharge dates, and survival time. Information was gathered on whether PCT testing and other diagnostics were performed, as well as antibiotic and antiviral administration. Data for all patients 16 years old or over who were admitted to hospital between 1 February 2020 and 30 June 2020 and who had a confirmed positive PCR COVID-19 test during this period were eligible for the study.^5^

Total length of stay was calculated as the days between either the date of positive COVID-19 test or the date of hospital admission, whichever was later, and hospital discharge date. Length of ICU stay was calculated as the days between either a positive COVID-19 test result or ICU admission date, whichever was later, and ICU discharge date. General ward length of stay was found by subtracting ICU length of stay from total length of stay. After propensity score matching (PSM), participants with missing total or ICU length of stay were excluded. In addition, observations where the ICU length of stay was greater than the total length of stay or where either was greater than survival time were assumed to be erroneous and discarded.

Treatment was defined as having PCT tested at baseline, defined as the day of the first positive sample for COVID-19 (±1 day). Balance of important confounders between treatment and control groups was achieved using PSM. Full details of this procedure are available in our companion paper.^9^ Patients were matched on age, sex, ethnicity, number of comorbidities, smoking status, index of multiple deprivation decile, quick SOFA (qSOFA), national early warning score 2 (NEWS2), confusion, uraemia, respiratory rate, blood pressure, age >65 score (CURB-65), 4C mortality score for COVID-19, early secondary bacterial infection, admission to ICU at baseline, baseline lung imaging category, the logarithms of baseline CRP level, neutrophil count, white cell count, D-dimer and troponin and indicator variables denoting missing blood test data.

### Model structure

**Figure 1.**
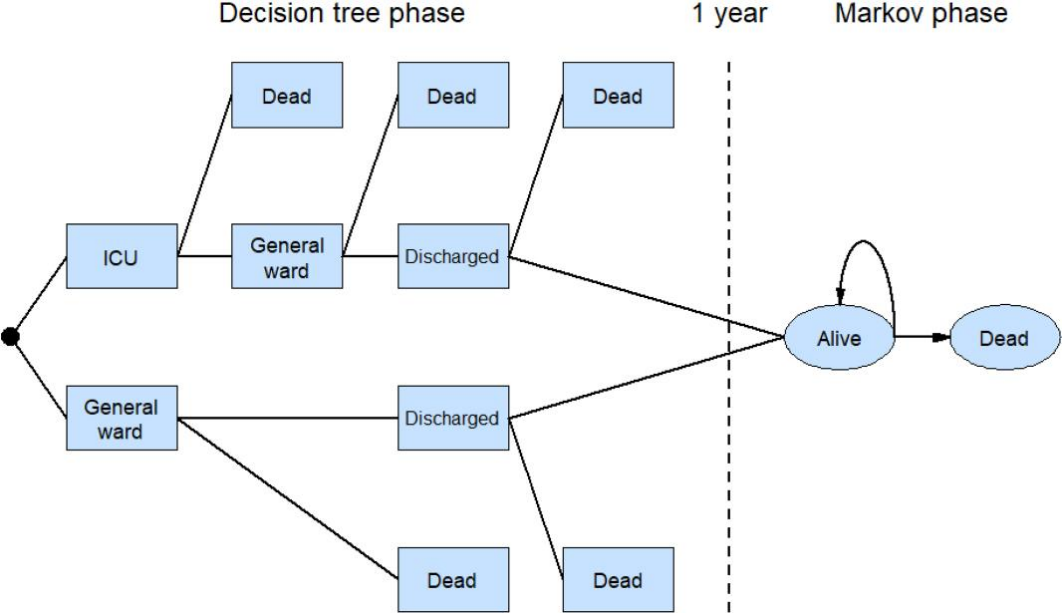
Economic evaluation model. Patients who did and did not receive a PCT test followed the same pathway. This figure appears in colour in the online version of *JAC* and in black and white in the print version of *JAC*.

The model structure is shown in Figure 1. As with several previous studies of COVID-19-related interventions,^10–12^ the model had two phases. The first was a decision tree representing the acute phase following hospitalization with COVID-19. Patients’ PCT levels were either initially tested or not. Hospitalization could either be in a general ward or ICU, or a mixture of both over the course of a patient’s stay. Patients either died in hospital or were discharged. The decision tree phase had a time horizon of 1 year. Patients still alive at 1 year then entered a Markov phase with a lifetime horizon. This had two states: alive and dead, with the latter being an absorbing state. Results are reported separately for the decision tree phase alone and both decision tree and Markov phase combined.

### Utilities

Quality-of-life data were not collected from patients, so previously published values were used to represent their utility. A review of the literature^13^ revealed a paucity of relevant data on quality of life for people hospitalized with COVID-19. Baseline utilities were calculated using the age/sex-specific UK population norms used by McNamara *et al*.,^14^ and in line with previous studies^15^ each day in a general ward/ICU was assigned a utility decrement. The disutility for general ward was taken from Wilcox *et al*.^16^ and that for ICU was taken from Hollman *et al*.^17^ Each day of antibiotic treatment was also assigned a decrement, taken from Oppong *et al*.,^18^ representing the potential for complications. Utility decrements are summarized in Table 1.

**Table 1.** Utility decrements and costs by component of COVID pneumonia admission pathway

	Utility decrement		Cost (£)	
	Value	Source	Value	Source
General ward	−0.36	Wilcox *et al*.^16^	£487.50 per day	Metry *et al*.^19^
ICU	−0.58	Hollmann *et al.*^17^	£2386 per day	NHS Reference Costs^20^
PCT			£15.20 per test	NICE^21^
Antibiotics	−0.05	Oppong *et al*.^18^	Varies, see methods	eMIT;^22^ BNF^23^
AMR			£2.12 per prescription	Oppong *et al*.^24^

### Costs

The daily cost of a general ward stay was taken from NICE guidance on economic evaluation for COVID-19 therapeutics.^19^ The latest figures were for 2019/20, so an inflation uplift of 2.5% was applied.^25^ The daily cost of an ICU stay was obtained from NHS reference costs for clinical care for 2020/21.^20^

The average unit price for PCT testing has previously been estimated by NICE, based on list prices of the tests and no discounts assumed. This estimate incorporates overhead costs, including capital, service and maintenance, and calibration costs.^21^ As the cost estimate was for 2015/16, an inflation uplift of 10.4% was applied.^20^

To calculate the cost of antibiotics in our study, data on the name, dose and frequency of dose for antibiotics were collected. These data were interpreted with the assistance of a clinician. Data on names of prescribed antibiotics were provided in our dataset in two ways: as coded types of antibiotics for common types of antibiotic; and as free text. Inspection of the data showed that in many cases, the free text contained various different spellings of, shorthand versions of, and typographical errors in, names of both coded antibiotics and non-coded but common antibiotic types. It was furthermore established that some instances of free text referred to prescribed medication that was not an antibiotic.

As a result of this, regular expressions were used, where possible, to correct data contained in free text to standardized names of antibiotics. This reduced the number of unique values in the free text from 195 to 57. After removal of 19 non-antibiotics from this list, and merging with 49 coded antibiotic names, 63 unique valid antibiotics remained.

Information on dose was also provided as free text. Regular expressions were again used to ensure that, as far as possible, numeric and dose measures were consistently coded—for instance, reformatting ‘500 millilitres’ as ‘500 mL’.

Data on antibiotic name and dose were used to match observations in our dataset to publicly available data sources. As per NICE guidelines, medication was preferentially matched to a cost provided in the drugs and pharmaceutical electronic market information tool (eMIT)^22^ and, where this was not possible, to NHS indicative prices provided by NICE BNF records.^23^ An iterative process was followed to merge antibiotic records to these two datasets in order to correct idiosyncratic errors, and allow for instances where the prescribed dose was apparently unavailable but was present as a multiple of a recorded available dose in this costing data. This allowed the costing of 97.3% of antibiotic records in our data.

Excessive and inappropriate antibiotic administration raises the risk of AMR, with implication for future health expenditures. A per-dose cost representing the cost of AMR was estimated based on the method reported by Oppong *et al*.^24^

### Transition probabilities

Transition probabilities for the decision-tree phase were estimated from patient data. For the Markov phase, age/sex-specific transition probabilities were taken from Office for National Statistics national life tables.^26^

### Discounting

Utilities in the Markov phase were discounted at an annual rate of 3%, in line with NICE guidelines.^27^

### Bootstrapping

Point estimates of quality-adjusted life years (QALYs), costs and incremental cost-effectiveness ratios (ICERs), including and excluding the Markov phase, were calculated using the whole dataset. Bootstrapping with 100 000 iterations was then used to generate 95% CIs. The bootstrapping results were also used to construct cost-effectiveness acceptability curves (CEACs) by estimating the probability of baseline PCT being cost-effective at cost-per-QALY thresholds between £0 and £50 000.

### Robustness tests

Initial inspection of the data revealed that a number of individuals in the ‘no PCT’ group spent an entire year in the general ward, whereas the longest general ward stay in the PCT group was 158 days. As a robustness test, the bootstrapping analysis was repeated after removing those outliers.

## Results

Data from 6173 individuals with a positive COVID-19 test were collected. After quality control (e.g. removal of individuals with a COVID-19 test date outside of the study timeline, removal of those with inconsistent hospital admission/discharge dates), 6089 remained. Data from 5960 of 6089 (97.9%) people were used for the propensity-score-matched primary analysis, of whom 1548 (26.0%) had PCT tested at baseline and 4412 (74.0%) did not. This quality control process and matched analyses were conducted by the statistics team (D.G., P.P., R.W.) and are reported in more detail elsewhere.^9^

Due to missing data or inconsistencies in variables key to the health economic analysis, some further exclusions were necessary. Of those included in the primary matched analysis (*n* = 5960), 47 observations had missing ICU length of stay, 78 had missing total length of stay, and 38 were missing survival time. In addition, there were five cases in which ICU length of stay was longer than total length of stay, and 38 where total length of stay was greater than survival time. After these exclusions, there were 5771 people included in the analysis, of whom 1509 (26.1%) had PCT tested at baseline and 4262 (73.9%) did not. Table 2 summarizes the participants’ characteristics before and after these exclusions.

**Table 2.** Participant characteristics, weighted using propensity score-matching weights

		All participants in original matched analysis (*n* = 5960)				Participants included in health economic analysis (*n* = 5771)			
		Baseline PCT		No baseline PCT		Baseline PCT		No baseline PCT	
		Mean (SE)	Median (IQR)	Mean (SE)	Median (IQR)	Mean (SE)	Median (IQR)	Mean (SE)	Median (IQR)
Age (years)		70.0 (0.425)	73.0 (25.0)	72.4 (0.249)	76.0 (22.0)	70.0 (0.429)	72.5 (25.0)	70.1 (0.597)	71.5 (25.0)
Sex, %	Male	56.4 (1.26)		55.3%(0.749)		56.4 (1.28)		58.6 (1.77)	
Ethnicity, %	White	79.3 (1.03)		76.6 (0.637)		79.1 (1.05)		80.6 (1.36)	
	Black	4.13 (0.506)		1.95 (0.208)		4.24 (0.519)		3.77 (0.777)	
	Asian	6.78 (0.639)		3.08 (0.260)		6.83 (0.649)		5.65% (0.83)	
	Mixed	0.711 (0.214)		0.793 (0.134)		0.663 (0.209)		1.03 (0.421)	
	Other	3.36 (0.458)		4.28 (0.305)		3.38 (0.465)		3.58 (0.552)	
IMD decile		4.13 (0.0734)	4.00 (5.00)	4.50 (0.0452)	4.00 (5.00)	4.14 (0.0745)	3.50 (5.00)	4.14 (0.107)	2.50 (6.00)
Number of comorbidities		1.99 (0.0491)	2.00 (3.00)	2.65 (0.0297)	2.00 (3.00)	1.99 (0.0493)	1.50 (3.00)	2.06 (0.0546)	1.50 (2.00)
4C mortality score for COVID-19		9.73 (0.0967)	10.0 (5.00)	9.80 (0.0576)	10.0 (4.00)	9.73 (0.0981)	9.50 (5.00)	9.85 (0.137)	9.50 (6.00)
*N*		5960				5771			

Means for included participants weighted using propensity score-matching weights. SE, standard error.

Table 3 gives decision-tree transition probabilities. The probability of transitioning from hospitalized to discharged was a little over two-thirds, with a 0.008 lower probability for people administered PCT at baseline. Conditional on being discharged, there was a probability of around 0.6 of surviving to 1 year and entering the Markov phase. This probability was 0.1 higher for people administered PCT at baseline.

**Table 3.** Decision-tree phase transition probabilities for patients who had a PCT performed at baseline and those who did not

Transitions		Probabilities	
From state	To state	PCT at baseline	No PCT at baseline
Hospitalized	Dead	0.309	0.301
	Discharged	0.691	0.699
Discharged	Dead	0.385	0.395
	Markov phase	0.615	0.605

**Figure 2.**
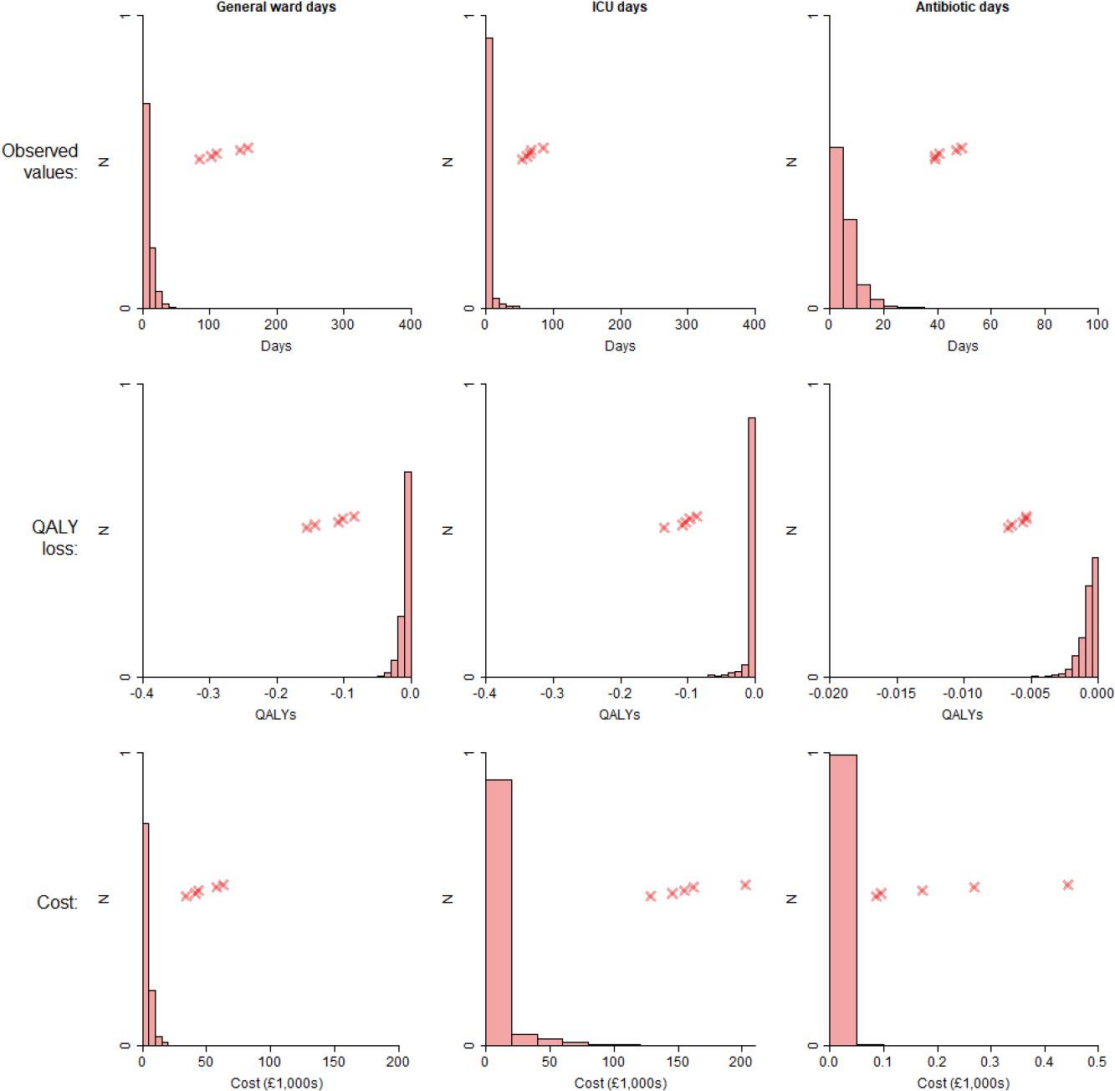
Weighted histograms of ward/ICU stays and antibiotic days for patients receiving a PCT test at baseline, and associated QALY losses and costs. Crosses indicate the five highest values. This figure appears in colour in the online version of *JAC* and in black and white in the print version of *JAC*.

**Figure 3.**
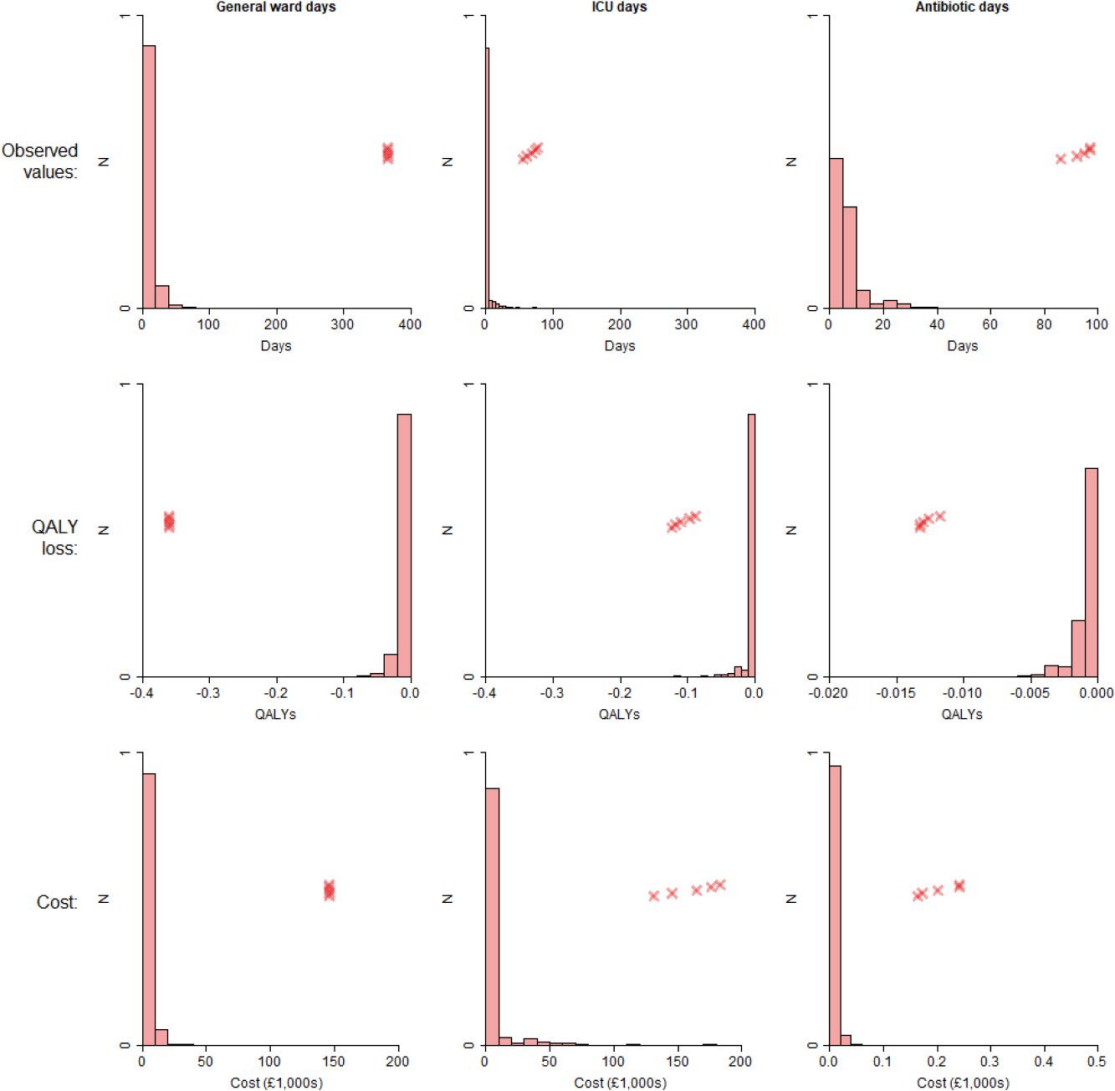
Weighted histograms of ward/ICU stays and antibiotic days for patients not receiving a PCT test at baseline, and associated QALY losses and costs. Crosses indicate the five highest values. This figure appears in colour in the online version of *JAC* and in black and white in the print version of *JAC*.

Table 4 shows the average days spent in a general ward and in ICU, days on antibiotics, and number of PCT tests, along with associated QALY losses and costs. People who had a PCT test at baseline had shorter general ward stays, as well as shorter ICU stays, and spent less time on antibiotics, though note the considerable overlap in 95% CIs in each case. The ‘baseline PCT’ group received 1.5 more PCT tests on average compared with the ‘no PCT’ group. The biggest QALY losses were associated with general ward days, and the greatest costs were associated with general ward and ICU days, with these being orders of magnitude greater than the impacts of PCT testing and antibiotic treatments. For example, in the ‘baseline PCT’ group, the QALY loss associated with being in a general ward was −9.15 × 10^−3^, which is 11 times greater than the loss of −8.45 × 10^−4^ associated with being on antibiotics. Similarly, in the ‘no baseline PCT’ group, the average cost of their ICU stay was £6400, which is 604 times greater than the £10.60 cost for PCT testing. Figures 2 and 3 show the distribution of general ward/ICU length of stay, and of antibiotic days, along with the associated QALY losses and costs. There are long tails in general ward and ICU distributions, with the extreme outliers being more prevalent in the ‘no baseline PCT’ group than in the ‘baseline PCT’ group.

**Table 4.** Mean length of stay, PCT tests and antibiotics for patients who had a PCT performed at baseline and those who did not

	Baseline PCT		No baseline PCT		Baseline PCT		No baseline PCT		Baseline PCT		No baseline PCT	
	Mean	95% CI	Mean	95% CI	Mean QALY loss	95% CI	Mean QALY loss	95% CI	Mean cost	95% CI	Mean cost	95% CI
General ward days	9.28	(8.85–9.69)	10.7	(9.81–11.5)	−0.00915	(−0.00955 to −0.00872)	−0.0105	(−0.0114 to −0.00967)	£3710	(£3540–£3870)	£4270	(£3920–£4610)
ICU days	2.55	(2.24–2.85)	2.68	(1.96–3.28)	−0.00404	(−0.00452 to −0.00355)	−0.00426	(−0.00521 to −0.00311)	£6070	(£5330–£6790)	£6400	(£4670–£7820)
PCT tests	2.24	(2.10–2.37)	0.694	(0.495–0.859)					£34.10	(£32.00–£36.10)	£10.60	(£7.53–£13.10)
Antibiotic days	5.94	(5.71–6.17)	6.78	(6.37–7.16)	−0.000814	(−0.000845 to −0.000781)	−0.000927	(−0.000980 to −0.000873)	£5.56	(£4.90–£6.14)	£4.85	(£4.44–£5.26)
AMR									£5.61	(£5.41–£5.81)	£6.14	(£5.83–£6.44)

**Figure 4.**
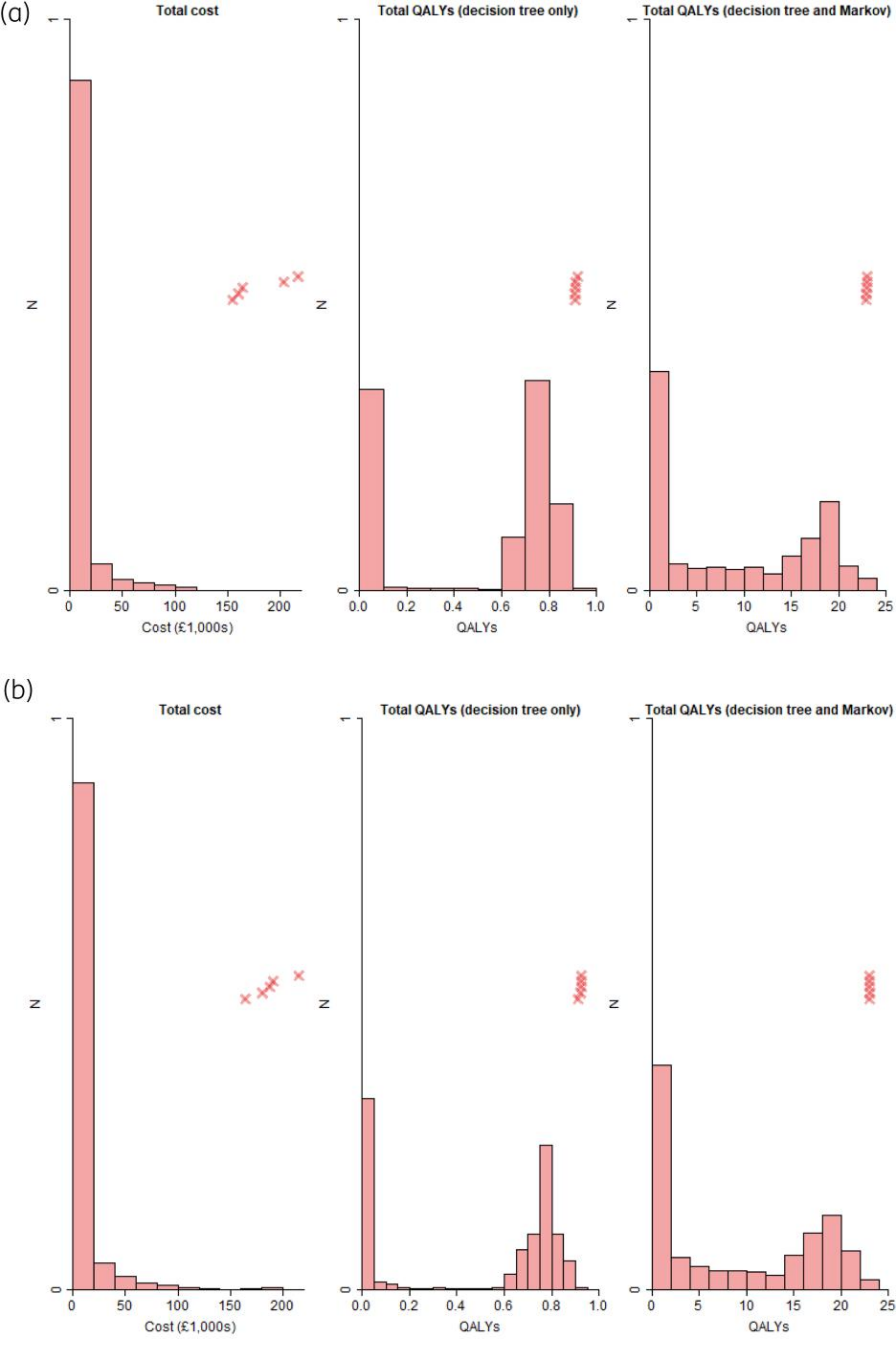
Weighted histograms of total cost and QALYs. Crosses indicate the five highest values. (a) Participants with PCT tested at baseline. (b) Participants without PCT tested at baseline. This figure appears in colour in the online version of *JAC* and in black and white in the print version of *JAC*.

In Table 5, the average survival time (capped at 365.25 days) was 2 days higher for people who had PCT testing at baseline, at 234 days, though there was considerable overlap in 95% CIs. The baseline utility of each group was similar (0.767 versus 0.769), but people who had PCT testing at baseline accrued more QALYs, both when considering the decision-tree phase alone (0.486 versus 0.479) and when also including the Markov phase (8.76 versus 8.62). The total cost was also lower when people had a PCT test performed at baseline (£9830 versus £10 700). Figure 4 shows the distributions of total costs and QALYs, both including and excluding the Markov phase. For total costs, there was a long tail, with 90% of patients’ costs less than £15 000, yet at the same time, over 50 patients had costs in excess of £100 000, and the very highest costs were over £200 000. The distributions of QALYs were bimodal, with peaks for the decision tree alone occurring between 0 and 0.1 QALYs, and between 0.7 and 0.8 QALYs. When including the Markov phase as well, peaks were observed between 0 and 2 QALYs, and between 18 and 20 QALYs.

**Table 5.** Survival time, total QALYs and costs for patients who had a PCT performed at baseline and those who did not

	Baseline PCT		No baseline PCT	
	Mean	95% CI	Mean	95% CI
Survival time (days)	234	(227–241)	232	(222–241)
Probability of 1 year survival	0.615	(0.596–0.634)	0.605	(0.578–0.630)
Baseline utility	0.767	(0.765–0.769)	0.769	(0.766–0.772)
Total QALYs (decision-tree phase only)	0.486	(0.472–0.501)	0.479	(0.460–0.498)
Total QALYs (decision-tree and Markov phases)	8.76	(8.44–9.09)	8.62	(8.15–9.08)
Total cost (£)	9830	(9040–10 600)	10 700	(8830–12 300)
ICER (decision-tree phase only)			−117 000	(−1 300 000 to 1 180 000)
ICER (decision-tree and Markov phases)			−5930	(−58 300 to 55 300)

**Figure 5.**
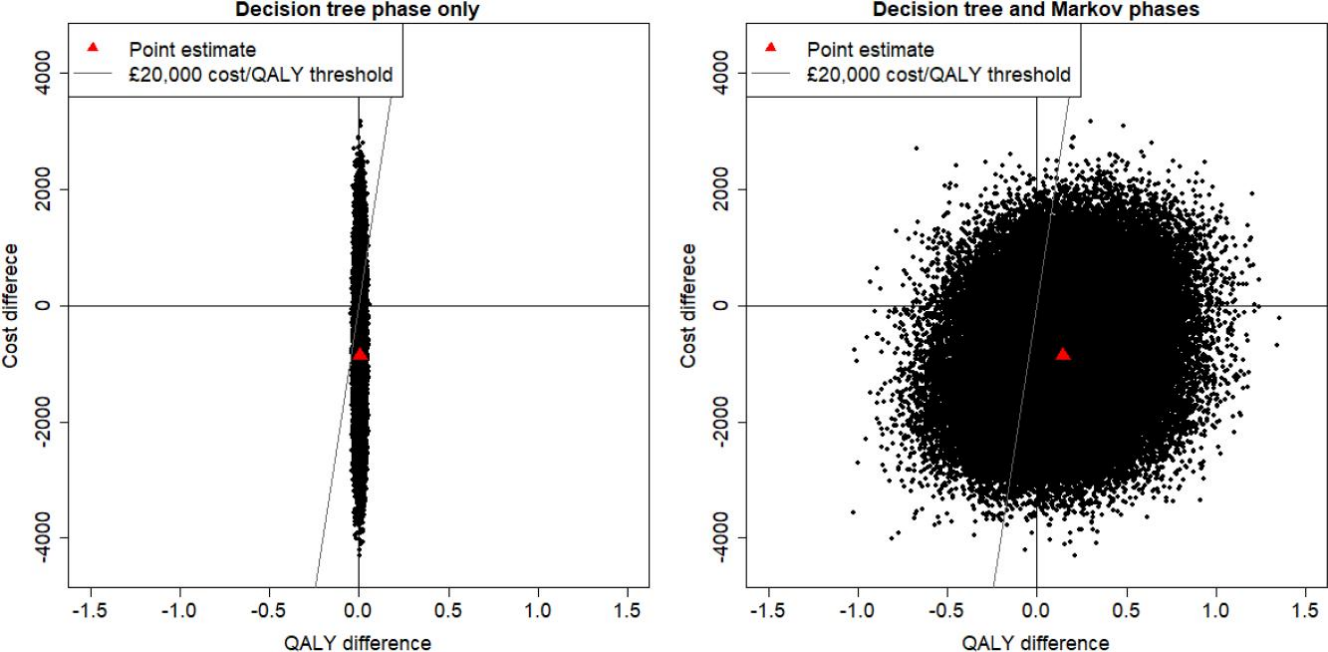
Cost-effectiveness planes. The *x*-axis and *y*-axis show, respectively, the QALY and cost differences between patients given and not given PCT tests at baseline with a 1 year (left) and lifetime (right) horizon. This figure appears in colour in the online version of *JAC* and in black and white in the print version of *JAC*.

**Figure 6.**
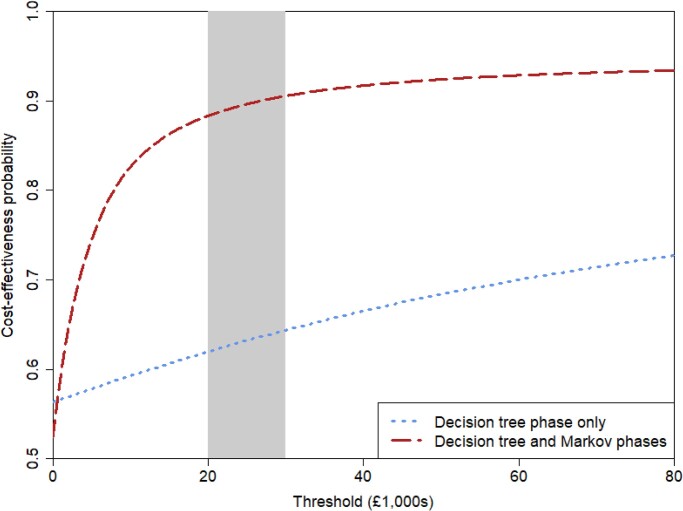
Cost-effectiveness acceptability curve. This figure appears in colour in the online version of *JAC* and in black and white in the print version of *JAC*.

As baseline PCT testing resulted in more QALYs and lower costs, the point estimate of our analysis implies that baseline PCT testing is a dominant strategy against no baseline PCT testing. However, Figure 5 puts the point estimate in context by illustrating the results of the bootstrap analysis, demonstrating that there is a large amount of uncertainty around that conclusion. Figure 6 shows the CEACs, and with a cost-per-QALY threshold of £20 000 the probability of cost-effectiveness is 0.579 when considering only the decision-tree phase, which rises to 0.872 when considering both decision-tree and Markov phases.

There were 13 patients who spent the entire year of the decision-tree phase in a general ward in the ‘no baseline PCT’ group. After repeating the analysis with these outliers removed, the probability of baseline PCT testing being cost-effective rose to 0.753 when considering a 1 year horizon, and to 0.862 when considering a lifetime horizon. Full results are provided in Tables S1 and S2 and Figures S1 and S2 (available as Supplementary data at *JAC* Online).

## Discussion

The results of this cost-effectiveness analysis demonstrate that using PCT to guide antibiotic decisions in individuals hospitalized with COVID-19 is more likely to be cost-effective than not, based on a cost-per-QALY threshold of £20 000, with a considerable amount of uncertainty in this conclusion, however. Baseline PCT testing resulted in more QALYs and lower costs overall both in the short (1 year post-admission) and long-term (lifetime analysis). However, a key driver of uncertainty in this analysis is the estimated QALYs; as an analysis largely based on matched retrospective observational data, we did not have quality-of-life data directly available and had to rely on estimates from the literature for this crucial component of the model.

There have been some published studies that have looked at the impact of using PCT to guide antibiotic therapy in patients hospitalized with COVID-19, all consistently concluding that PCT is a safe and simple way to reduce antibiotic use in patients hospitalized with COVID-19.^28–31^ On the other hand, the usefulness of PCT has been questioned when applied to patients treated with contemporary state-of-the-art immunomodulators.^32^ To our knowledge, this is the first study to examine whether the use of PCT to guide antibiotic prescribing in patients hospitalized with COVID-19 is a cost-effective strategy. Van der Pol *et al*.^33^ conducted a systematic review of economic analyses of diagnostics for respiratory tract infections and found three studies that evaluated the cost-effectiveness of PCT testing in a hospital setting (two studies focused on hospital in general and one on intensive care).^33^ All three of these studies concluded that PCT was likely to be cost-effective, but none of the studies were from a UK perspective.^34–36^ There are currently two randomized controlled trials in the UK in adults evaluating the use of PCT in individuals with sepsis: ADAPT-Sepsis in hospitalized adults (nearing completion); and PRONTO in adults with suspected sepsis presenting to the Emergency Department (recruitment completed).^37,38^ Both of these studies include planned health economics analysis, and it will be useful to compare their results with the findings from the current study.

Comparing the findings reported here with the companion paper of Sandoe *et al*.,^9^ they are consistent, as should be expected given that they both use the same data. Their main result was significantly fewer antibiotic days for those who had a PCT test at baseline compared with those who did not, which is also shown here. Sandoe *et al*. do not report statistically significant differences in length of stay and mortality (at 30 and 60 days) whereas here differences in those variables are key drivers in the central estimate that baseline PCT testing is cost-effective. The results are not contradictory, and in both analyses, there are similar differences in the point estimates of the variables, but we do not test for statistical significance between those variables, as such tests are not relevant for our analytical approach. It is also possible for small and non-significant differences in a variables such as ICU length of stay to lead to greater differences in costs and QALYs due to the associated large unit costs and disutilities.

A key strength of our study is that it is based on ‘end-to-end’ individual-level data for a large, multiregional cohort of patients rather than having to rely heavily on a linked evidence approach, which is common for the economic evaluation of diagnostic tests. This limited the number of modelling assumptions that needed to be made to build the model, and means that the evidence on which we have based the model is reflective of real-world clinical practice. As an observational study, however, there is a risk that some unknown confounding factors may have influenced effectiveness estimates, which could not be adjusted for in the matched analysis. A sensitivity analysis conducted as part of the main statistical analysis indicated that this possibility of residual confounding cannot be ruled out.^9^ The retrospective nature of the data collection also meant that we did not have individual-level quality-of-life data available, and we had to rely on generic utility decrements associated with being in general or ICU wards, and being on antibiotics, leading to high uncertainty in the incremental QALYs. This study was conducted in England and Wales, and judged cost-effectiveness according to the relevant Health Technology Assessment body for those countries, i.e. NICE. Thus its findings will not necessarily translate to other contexts where not only may the patient population have different characteristics, but where different standards may be used for assessing cost-effectiveness.

This economic evaluation, based on a large cohort of retrospective matched observational individual-level data from 11 NHS Trusts and Health Boards in England and Wales, provides real-world evidence that using PCT to guide antibiotic therapy in patients hospitalized with COVID-19 is more likely to be cost-effective than not, albeit with considerable uncertainty.

Future work could usefully look at other respiratory diseases. Also, given that the mean ICU stay was relatively short, it could be that baseline PCT testing is more cost-effective in diseases with longer average ICU stays, and future research could address this. This study focused specifically on PCT testing at baseline, i.e. at the time when a positive COVID-19 result was returned. This means that many people in the control group had a PCT test at some point, and may well have ceased/not initiated antibiotic treatment on the basis of the result. The reason for choosing baseline testing as the treatment/control criteria is that baseline PCT testing represents a clear, implementable protocol for hospitals. Future work could usefully explore the (cost-) effectiveness of PCT testing later in the treatment pathway.

## Supplementary Material

dkae167_Supplementary_Data

## Transparency declarations

J.A.T.S. has received research funding from MRC, EPSRC, NIHR, Lumos diagnostics, and Jon Moulton Charity Trust in relation to development of infection diagnostics and is a council member of the British Society for Antimicrobial Chemotherapy (BSAC); is co-investigator (co-I) for PROTECT (NIHR-HTA 156664); has received honoraria from Medtronic and Tillotts Pharma. M.A. has received honoraria payments for lectures and research from Shionogi, Merck and Pfizer and received research funding from The Showering Fund. P.D. is Chief investigator (CI) for the ADAPT-Sepsis Trial (NIHR-HTA 15/99/02) and co-I for PROTECT (NIHR-HTA 156664). J.E. is co-I for PROTECT (NIHR-HTA 156664). T.H. is CI for the RISC-Sepsis Study (NIHR EME 128374) and CI for the SHORTER trial (NIHR HTA 134101). I.J.M. is co-I on the RISC-Sepsis Study (NIHR EME 128374). D.P. is British Infection Association President. N.P. has received honoraria payments for lectures from Thermo Fisher. D.S. is co-I on RISC-sepsis (NIHR EME 128374). T.S. has received fees from Thermo Fisher Ltd in relation to PCT testing in the ICU and is CI for VAMp-SEPSIS (HCRW 1951/2023) and co-I on the EXTEND trial (NIHR 131784). S.T. is co-CI for PRONTO (NIHR HTA 17/136/13) and co-I for PROTECT (NIHR-HTA 156664). E.T.J. is co-I for PRONTO (NIHR HTA 17/136/13), BATCH (NIHR-HTA 15/188/42) and PROTECT (NIHR-HTA 156664). E.D.C. is CI for the BATCH Trial (NIHR HTA 15/188/42), co-CI for the PROTECT study (NIHR HTA 156664), co-I for the PRONTO Trial (NIHR HTA 17/136/13), a former member of the NICE diagnostic advisory committee (2014–20) and NICE sepsis guideline development committee (2014–16). P.P. is co-I for PRONTO (NIHR HTA 17/136/13) and co-CI for PROTECT (NIHR HTA 156664). L.B.H. is co-I for PRONTO (NIHR HTA 17/136/13) and BATCH (NIHR HTA 15/188/42). All other authors declare no competing interests.

## Supplementary data

Figures S1 and S2 and Tables S1 and S2 are available as Supplementary data at *JAC* Online.
