# Supplementary material for: The cost-effectiveness of procalcitonin for guiding antibiotic prescribing in individuals hospitalized with COVID-19: part of the PEACH study
Source: J Antimicrob Chemother. 2024 Jun 6;79(8):1831–42. doi: 10.1093/jac/dkae167 (PMC11290882; doi:10.1093/jac/dkae167)
Supplement: dkae167_Supplementary_Data [file dkae167_supplementary_data.docx]

**Supplementary online material – robustness test removing outliers**

**Table S1: Mean length of stay, procalcitonin tests and antibiotics for patients who had a PCT performed at baseline and those that did not**

|  | Baseline PCT | | No baseline PCT | | Baseline PCT | | No baseline PCT | | Baseline PCT | | No baseline PCT | |
| --- | --- | --- | --- | --- | --- | --- | --- | --- | --- | --- | --- | --- |
|  | Mean | 95% CI | Mean | 95% CI | Mean QALY loss | 95% CI | Mean QALY loss | 95% CI | Mean cost | 95% CI | Mean cost | 95% CI |
| General ward days | 9.28 | (8.85 - 9.69) | 10.6 | (9.06 - 11.7) | -0.00915 | (-0.00955 - -0.00872) | -0.0104 | (-0.0116 - -0.00893) | £3710 | (£3540 - £3870) | £4230 | (£3620 - 4690) |
| ICU days | 2.55 | (2.24 - 2.84) | 3.1 | (2.17 - 3.89) | -0.00404 | (-0.00452 - -0.00355) | -0.00492 | (-0.00618 - -0.00345) | £6070 | (£5330 - £6790) | £7390 | (£5180 - 9290) |
| PCT tests | 2.24 | (2.10 - 2.37) | 0.738 | (0.498 - 0.936) |  |  |  |  | £34.10 | (£32.00 - 36.10) | £11.20 | (£7.58 - 14.20) |
| Antibiotic days | 5.94 | (5.71 - 6.17) | 6.84 | (6.36 - 7.28) | -0.00081 | (-0.000845 - -0.000782) | -0.00094 | (-0.000996 - -0.000871) | £5.56 | (£4.91 - 6.14) | £5.95 | (£4.92 - 6.75) |
| AMR |  |  |  |  |  |  |  |  | £5.61 | (£5.40 - 5.81) | £6.23 | (£5.80 - 6.62) |
| *Note.* 13 participants in no baseline PCT group with year-long ward lengths of stay removed; PCT=procalcitonin; CI=confidence interval; ICU=intensive care unit; AMR=antimicrobial resistance | | | | | | | | | | | | |

**Table S2: Survival time, total quality of life and costs for patients who had a PCT performed at baseline and those who did not**

|  | Baseline PCT | | No baseline PCT | |
| --- | --- | --- | --- | --- |
|  | Mean | 95% CI | Mean | 95% CI |
| Survival time (days) | 234 | (227 - 240) | 230 | (221 - 239) |
| Probability of 1 year survival | 0.615 | (0.596 - 0.634) | 0.599 | (0.572 - 0.625) |
| Baseline utility | 0.767 | (0.765 - 0.769) | 0.768 | (0.765 - 0.772) |
| Total QALYs (decision tree phase only) | 0.486 | (0.472 - 0.501) | 0.476 | (0.457 - 0.495) |
| Total QALYs (decision tree and Markov phases) | 8.76 | (8.44 - 9.08) | 8.65 | (8.17 - 9.11) |
| Total cost (£) | 9830 | (9040 - 10600) | 11600 | (8980 - 13900) |
| ICER (decision tree phase only) |  |  | -171000 | (-1900000 - 1760000) |
| ICER (decision tree and Markov phases) |  |  | -15200 | (-106000 - 104000) |
| *Note*. 13 participants in no baseline PCT group with year-long ward lengths of stay removed; PCT=procalcitonin; CI=confidence interval; QALY=quality adjusted life year; ICER=incremental cost effectiveness ratio | | | | |


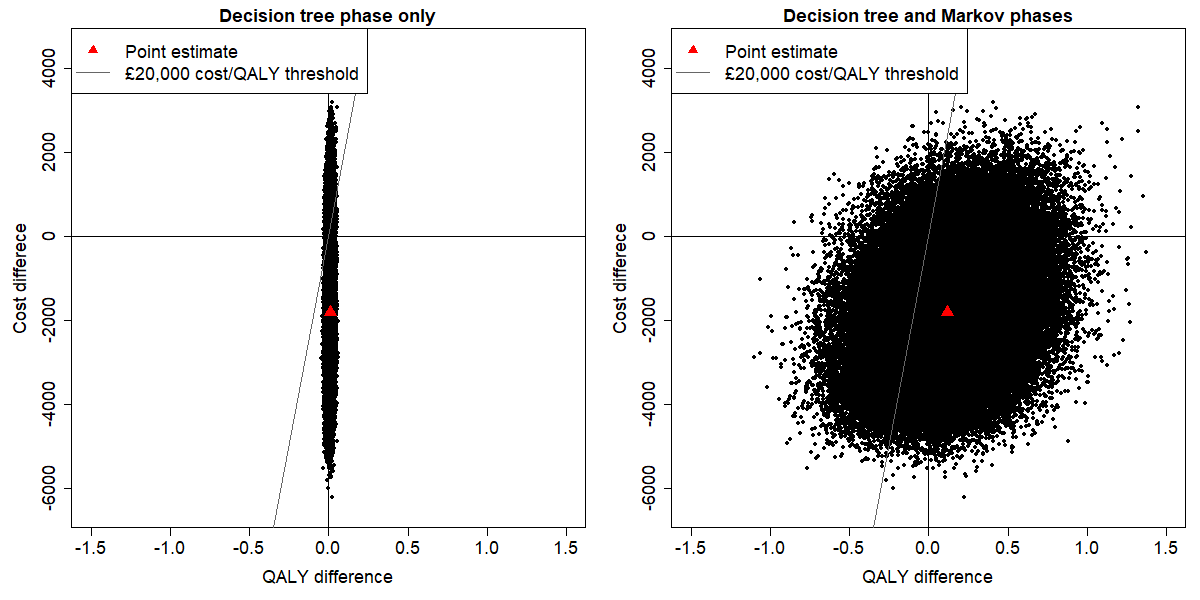


Figure S1: Cost effectiveness planes. The x- and y- axes show respectively the QALY and cost differences between patients given and not given procalcitonin tests at baseline with a one-year (left) and lifetime (right) horizon; 13 participants in no baseline PCT group with year-long ward lengths of stay removed; QALY=quality adjusted life year


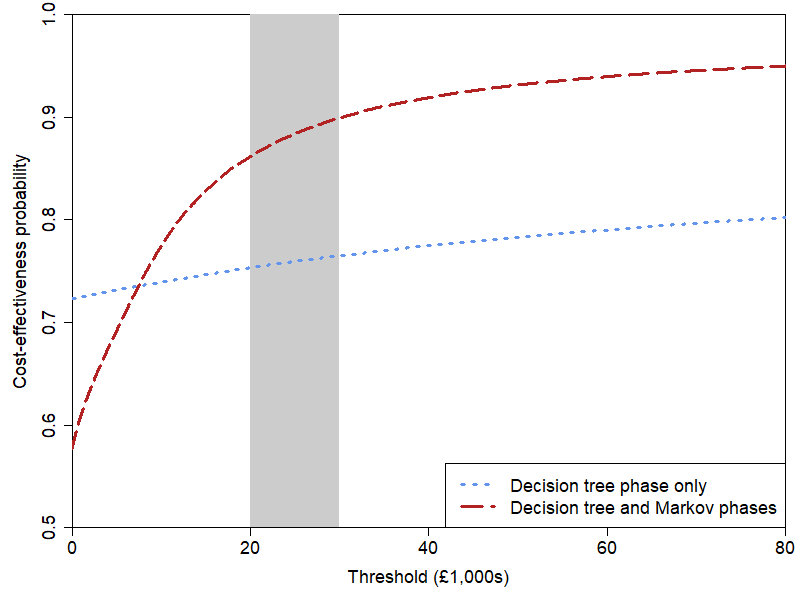


Figure S2: Cost effectiveness acceptability curve. 13 participants in no baseline PCT group with year-long ward lengths of stay removed
